# The Week's Work

**Published:** 1910-11-05

**Authors:** 


### The Week's Work.

### KING EDWARD MEMORIALS.

While in a certain sense Mr. Asquith's dash of cold water on the scheme for a national memorial to the late King was undoubtedly beneficial in spurring local efforts to greater attempts than they would otherwise have visioned, it must not be forgotten that it has also practically closed the door on any concerted scheme which might have influenced committees supremely throughout the Empire. The model city plan, as suggested by Mr. Ebenezer Howard, is in the circumstances impossible, for no individual community, even so rich and enterprising as London, can undertake it and carry it through. Already there is a tendency to look for satisfaction in statues or simple plastic monuments erected in local squares or parks. Bristol, for example, has decided upon some such memorial, and it is desirable, therefore, that local committees should be made acquainted with the expressed wish of the King that memorials to his father should be of such a character as to perpetuate in some practical way the eminently practical interest which King Edward took in charitable work. Art memorials, however excellent, will not achieve that object; but there are many local schemes which, if carried out, will materially help to achieve it. Doubtless the decision of the Lord Mayor's committee will very largely influence other committees in England and abroad, and it is satisfactory to find that the members of this central committee are in no hurry to make up their mind. Much thought and careful consideration are required before any scheme can be accepted, and although the number of projects submitted is very large and the nature of them very varied, it is difficult to find in the published lists a single one that is quite free from objections on some score or other. The hospital world is well represented on the central committee, and its representatives may be looked to to consider any schemes affecting it as a whole conscientiously and carefully.

### SHEFFIELD ROYAL HOSPITAL.

A good deal of discussion took place at the recent annual meeting of the Sheffield Royal Hospital on the vexed question of recommendations and their alleged abuse by persons who could afford to pay. The Master-Cutler, Alderman G. Senior, who presided, thought "there were thousands of people who wrongfully took advantage of the Sheffield hospitals," and that this was due partly to "the indiscriminancy shown by some people in giving recommendations without investigating the cases properly." Mr. Philip K. Wake endorsed this view. Sir John Bingham, a vice-president of the institution, agreed that "the issue of recommendations should be looked into more closely." No one apparently suggested that the appointment of a hospital almoner would meet the case. We venture to commend to the above gentlemen what has been said on the value of almoners in The Hospital of July 2, page 417, and in proof of that value to read the detailed account of a lady almoner's work which appears on page 507 of The Hospital of July 23. Further than this, if "The lessons learnt in the out-patient department" (see The Hospital of July 30, page 535) are then studied, we have little doubt that Alderman Senior, Mr. Philip K. Wake, and Sir John Bingham will be in a position to understand, as well as to denounce, the abuses they complain of so fully as to make the remedy of these abuses fairly obvious, if not easy.

### HOSPITALS AND THE CORONATION.

Hospitals have been the inspiration of so many of the proposals to commemorate the late King, that they may claim to be favoured more intimately by English Sovereigns than perhaps any other social institutions. King George's first public appearance was made to inspect the London Hospital: the metropolitan institutions, therefore, should have their own relation to the Coronation next June. No better proposal for giving them this has been suggested at present than that put forward by a writer in the *Times*: "Owing to the shortness of the route of the forthcoming Coronation procession only those persons who can afford to pay fabulous prices for seats will be able to witness it. To enable those of limited means to participate, and at the same time to promote a deserving charity, I suggest that stands capable of holding large numbers be erected by the Government on all open spaces along the line of route, *i.e.* say along one side of Constitutional Road, and that half-a-crown be charged for admittance, the proceeds to be devoted to the King's Hospital Fund; to prevent speculation in seats, the people to pay at a turnstile in the order in which they arrive on the actual Coronation day." This suggestion is simple and practical, and requires little comment. We mention it here so that hospital secretaries, as a class, may be ready to give it general support when the time comes.

### THE LATE HENRI DUNANT.

Though more prominently identified with the work of the Red Cross, of which organisation he was practically the founder, the late M. Henri Dunant, whose death was announced last week, took a keen interest in hospital affairs, not only in his own country, Switzerland, but all over the world. He was a profound believer in his maxim that philanthropy knows no barriers of system, race, or creed, and an equally strong pleader for the internationalisation of hospital and, indeed, all charitable effort. Though not an authority on hospital management, he was yet conversant with the various systems in existence on the Continent and in this country, and was particularly struck with the excellent work that was being done under the voluntary system both in England and America. Voluntary effort, which had succeeded all along the line so far as the Red Cross was concerned, he held offered the best possible solution for many of the problems of charitable relief; but at the same time he was fully conscious of the fact that it meant the delegation to private enterprise of business that should be, to some extent at least, an affair of the community. In his later years he sometimes criticised the Swiss system, and held up the German example as one to be followed; but his main interest was obviously in the matters he had so close at heart—the humanisation, as he loved to call it, of war. Miss Nightingale's work he always regarded as excellent in the highest degree, and the initial efforts which he himself made in the late 'sixties of the last century were undoubtedly influenced by her good work in the Crimea. He lived to see the "illusions" and "fantastic ideals" that he cherished made the basis of a practical system which is now recognised by all civilised countries, so much so in fact that one of the tests of civilisation in a nation's management of a war lies in its observance of the tenets laid down by Dunant.

### A CASE FOR A CORONER.

With great regret it has to be recorded that by the alleged default of a resident medical officer the life of a child has been lost which might very possibly have been saved. This statement is based upon the report of an application to one of the stipendiary magistrates by a young man, the father of the infant. The magistrate declined to interfere with a matter in which he had, as he said, no jurisdiction and about which he had heard one side only; but the statement of the applicant is sufficiently explicit on the face of it to constitute a very serious indictment of the medical man concerned. According to the father's account his baby, aged sixteen months, fell suddenly ill, and on the third day of the illness a doctor, who saw the patient in the early hours of the morning, telephoned to the hospital asking them to receive the case and be prepared for immediate operation. The father arrived there at 8 a.m., and found the door locked; when he was admitted he discovered that nothing was ready, and the baby died in his arms six minutes later. If the disease was laryngeal diphtheria, and the contemplated operation was tracheotomy, it is fair to conclude that the delay may have meant the difference between life and death. It is also ascertained that the house-surgeon was just getting up, having made a mistake of half an hour in the time. On the facts as disclosed it may be suggested that an inquest should have been held on this case, and that the medical man who gave a certificate of death has been seriously at fault in not notifying the case to the coroner. It may be that the version set forth in the police-court is inaccurate in important respects; and it may be that the case was one of intussusception or other emergency, not diphtheria at all, in which case the wasted six minutes can have made no material difference. But the principle at stake is a serious one, and the lesson is one which all hospital medical officers must ever be mindful of.

### HOSPITAL SUNDAY IN HULL.

The committee of the Hull Hospital Sunday Fund is to be congratulated on the illustrated handbook it has issued, which is an interesting and instructive record of what has been done and summary of what is still to be achieved. On the title-page is a vignette portrait of the late Alderman Seaton, who, as early as 1874, founded Hospital Sunday in Hull, and on the next page is a spirited appeal to the citizens on behalf of the Fund, signed by the Mayor. Canon Lambert follows with a short and pointed article on the meaning of Hospital Sunday, which is illustrated by photographs of the late King and of Queen Alexandra. Then we have notes on the history of the local Royal Infirmary and of various other local hospitals, all well illustrated and written in a bright, readable manner. We commend page 31, dealing with King George's interest in the Fund, to other committees: it is a good example of legitimate advertisement in the cause of charity, a dignified appeal that points its own moral. Equally excellent is page 41, which contains a similar account of Queen Mary's interest in the work. Further on a short account of some interesting features in the city itself are given, a detailed description of the fine organ in the City Hall and a brief historical account of the rise of Hull being included. Nor must the special inset be omitted in the enumeration of the good things that this handbook contains. It is a neat little envelope, stamped with the words *Hospital Sunday, October* 30, 1910: *Special Effort to raise* £2,000 *as last year. Family collection*, with a space left blank underneath for the name and address of the subscriber. At the bottom is printed the following notice: "Please hand this envelope at the collection in your church or chapel or leave it in the box provided at any of the branch post offices of the city." This, we imagine, is an excellent way of appealing to the many who have never contributed to the Fund. It is an example that may well be followed by other committees. The Hull committee is evidently both enthusiastic, up-to-date, and practical, and we have not the slightest doubt that it will raise the fund it desires and surpass the figures of last year.

### THIS WEEK'S DRUG MARKET.

The main feature of the week's drug market is the advance in the price of cream of tartar, seidlitz powder, and tartarated soda, the common cause of these advances being the deficiency of the wine crops in France and Italy and the consequent scarcity of the crude cream of tartar, which is deposited during the fermentation of grape juice, and from the lees of wine. These articles have been advancing steadily in price for some months, and this week cream of tartar has further advanced several shillings per cwt., seidlitz powder 7s. per cwt., and tartarated soda 9s. per cwt., with probability of further advances. Tartaric acid is also tending dearer. On the other hand, citric acid is slightly cheaper. The price of quicksilver has again been reduced, and it is not improbable that makers of calomel, corrosive sublimate, and other salts of mercury may make a further slight reduction in their prices. Ergot of rye is dearer, as also is hydrastis. Cod-liver oil is slightly cheaper, but it is probable that when the winter demand sets in prices may advance somewhat. Potassium bromides and other salts of bromine are slightly dearer. Terebene is dearer. The opium market is quiet, and morphine and codeine are unchanged in price.

